# 

**Published:** 1896-06

**Authors:** 


### CONTENTS.

**ORIGINAL COMMUNICATIONS—**

PRACTICAL PATHOLOGY OF THE MUCOUS MEMBRANE OF THE NOSE, THROAT, AND EARS OF CHILDREN. By Thomas F. Rumbold, M.D., St. Louis, Mo. .......... 217

THE ACTION AND ADMINISTRATION OF CHLOROFORM. By Luther B. Grandy, M.D., Atlanta, Ga. .......... 223

ON THE DEFINITION OF HYPNOSIS. By Dr. Max Hirsch, Berlin .......... 232

PLACENTA PREVIA, AND REPORT OF CASES. By John R. Shannon, A.B., M.D., [illegible]aniss, Ga. .......... 237

**SOCIETY REPORTS—**

AMERICAN MEDICAL ASSOCIATION .......... 239

**CORRESPONDENCE—**

A DOUBLE MONSTROSITY .......... 263

**EDITORIAL—**

THE AMERICAN ACADEMY OF MEDICINE AND THE AMERICAN MEDICAL ASSOCIATION ... 264

**SELECTIONS AND ABSTRACTS—**

OBSTETRICS AND GYNECOLOGY .......... 271

SURGERY .......... 275

GENERAL MEDICINE AND THERAPEUTICS .......... 278

**MEDICAL ITEMS** .......... 283

**BOOK REVIEWS** .......... 287

**MEDICAL ITEMS—CONTINUED** .......... x, xi, xii, xiii, xiv, xv

**ADVERTISERS' INDEX TO ADVERTISEMENTS** .......... xvi

**CLASSIFIED INDEX TO ADVERTISEMENTS** .......... xviii

